# Evaluation of the performance of a multiplex reverse transcription polymerase chain reaction kit as a potential diagnostic and surveillance kit for rotavirus in Kenya

**DOI:** 10.1186/s40794-019-0087-7

**Published:** 2019-07-15

**Authors:** Cliff Odhiambo Philip, Margaret Koech, Nancy Kipkemoi, Ronald Kirera, Janet Ndonye, Abigael Ombogo, Mary Kirui, Erick Kipkirui, Brook Danboise, Christine Hulseberg, Stacey Bateman, Alexander Flynn, Brett Swierczewski, Esther Magiri, Elizabeth Odundo

**Affiliations:** 1 United States Army Medical Research Directorate-Africa, Nairobi, Kenya; 2 University of Michigan Medical School, Arbor, USA; 3 Center for Genome Sciences, US Army Medical Research Institute of Infectious Diseases, Frederick, Maryland USA; 4 Madigan Army Medical Center, Washington, USA; 5 Walter Reed Army Institute of Research, Silver Spring, USA; 6 Jomo Kenyatta University of Agriculture and Technology, Juja, Kenya

**Keywords:** Rotavirus, Diarrhea, EIA, PCR, RT-PCR, qRT-PCR, EPS

## Abstract

**Background:**

Diarrhea is a serious concern worldwide, especially in developing countries. Rotavirus is implicated in approximately 400,000 infant deaths annually. It is highly contagious elevating the risk of outbreaks especially in enclosed settings such as daycare centers, hospitals, and boarding schools. Reliable testing methods are critical for early detection of infections, better clinical management, pathogen surveillance and evaluation of interventions such as vaccines. Enzyme immunoassays have proved to be reliable and practical in most settings; however, newer multiplex reverse transcription polymerase assays have been introduced in the Kenya market but have not been evaluated locally.

**Methods:**

Stool samples collected from an ongoing Surveillance of Enteric Pathogens Causing diarrheal illness in Kenya (EPS) study were used to compare an established enzyme immunoassay, Premier™ Rotaclone® (Meridian Bioscience, Cincinnati, Ohio, U.S.A.), that can only detect group A rotavirus against a novel multiplex reverse transcription polymerase chain reaction kit, Seeplex® Diarrhea-V ACE Detection (Seegene, Seoul, Republic of Korea), that can detect rotavirus, astrovirus, adenovirus, and norovirus genogroups I and II. Detection frequency, sensitivity, specificity, turnaround time, and cost were compared to determine the suitability of each assay for clinical work in austere settings versus public health work in well-funded institutes in Kenya.

**Results:**

The Premier™ Rotaclone® kit had a detection frequency of 11.2%, sensitivity of 77.8%, specificity of 100%, turnaround time of 93 min and an average cost per sample of 13.33 United States dollars (USD). The Seeplex® Diarrhea-V ACE Detection kit had a detection frequency of 16.0%, sensitivity of 100%, specificity of 98.1%, turnaround time of 359 min and an average cost per samples 32.74 United States dollars respectively. The detection frequency sensitivity and specificity of the Seeplex® Diarrhea-V ACE Detection kit mentioned above are for rotavirus only.

**Conclusions:**

The higher sensitivity and multiplex nature of the Seeplex® Diarrhea-V ACE Detection kit make it suitable for surveillance of enteric viruses circulating in Kenya. However, its higher cost, longer turnaround time and complexity favor well-resourced clinical labs and research applications. The Premier™ Rotaclone®, on the other hand, had a higher specificity, shorter turnaround time, and lower cost making it more attractive for clinical work in low complexity labs in austere regions of the country. It is important to continuously evaluate assay platforms’ performance, operational cost, turnaround time, and usability in different settings so as to ensure quality results that are useful to the patients and public health practitioners.

## Background

Diarrheal disease is a leading cause of mortality and morbidity worldwide that places a considerable financial burden on health care systems and patients [1]. Though short-lived, the associated morbidity is significant [2], especially among children younger than the age of five where 1 in 9 child deaths worldwide is caused by diarrheal disease. This translates to 17% of all deaths in this age group which is more than deaths associated with acquired immune deficiency syndrome (AIDS), malaria and measles combined. Approximately two thirds of these deaths occur in sub-Saharan Africa and South Asia [3, 4]. Previous studies have indicated that the risk of infections and outbreaks is elevated among people working or living in enclosed communities such as nursing homes, hospitals, prisons, daycare centers, boarding schools, and military camps [5].

Among the enteric viruses, rotavirus stands out as the leading cause of infectious diarrhea in infants and children worldwide [6]. Each year, rotavirus causes approximately 25 million clinic visits, 2 million hospitalizations, and between 352,000 to 592,000 deaths (median: 440,000 deaths) in children < 5 years of age [7]. The virus is very stable in the environment and can remain infectious for weeks within raw food, treated and untreated water, which all represent possible sources of rotavirus gastroenteritis outbreaks [8].

Rotavirus disease imposes a heavy economic burden due to factors such as medical consultation and treatment as well as loss of time at work. Although rotavirus is generally the most common enteric pathogen in children worldwide, its role (and that of other enteric viruses) in many parts of Kenya is less understood due to limited resources and laboratory challenges in remote areas of the country. Rotavirus diarrhea is estimated to cause about 19–27% of all diarrhea hospitalizations of children < 5 years in Kenya where it is still the most common cause of severe gastroenteritis in children despite ongoing vaccination [9, 10]. It was noted in Peru that the rate of rotavirus infection and its associated disease burden is higher in the second year of life despite vaccine compliance. This observation indicates that protection by the vaccine may not be sustained beyond the first year of life in some populations [10–12].

Rotavirus is also known to cause diarrhea in older children and adults with severe illness occurring in immunocompromised hosts. Healthy adults and children who are carriers may not feel the disease burden, but they act as viable reservoirs and potential sources of outbreaks. Studies have shown that there is a high likelihood of back and forth infections between children and adults [13]. For these reasons, adults and healthy children (controls) who are part of EPS study were included in this study.

Laboratory diagnosis of rotavirus infection is usually performed using various techniques both conventional and molecular. Enzyme immunoassays (EIAs) are used as the standard test for rotavirus infection in many parts of the world [14]. Several polymerase chain reaction (PCR)-based protocols for the detection of human enteric viruses with higher sensitivities have been published, but only a few of them allow for simultaneous detection of the major enteric viruses in one assay. This is a necessary capability in order to understand the disease burden associated with viral gastroenteritis caused by multiple viruses circulating in Kenya [15, 16]. Diagnostic capabilities to detect a broad spectrum of diarrheal pathogens is lacking in many parts of the Kenya with many health care providers relying on empirical diagnosis without laboratory confirmation, a practice that can result in misdiagnosis and prescription of inappropriate treatment [4, 17, 18]. Clinical testing for rotavirus in the country is mostly done only in well-funded hospitals in urban areas, and is rarely done in remote regions where laboratories have insufficient resources. In places where testing is done, EIA and Reverse Transcription PCR (RT-PCR) are the most commonly applied techniques for rotavirus testing in Kenya [12, 19, 20].

A review of the epidemiology of human rotavirus associated with diarrhea in Kenyan children between 1975 to 2005 reported EIAs as the most commonly used testing platform followed by RT-PCR, polyacrylamide gel electrophoresis (PAGE), culture and fluorescence focus neutralization (FFN) respectively [21]. EIA was the screening platform of choice for the Rotavirus Vaccine Impact Evaluation in Kenya (RIPEK) study that was recently conducted by CDC Kenya, KEMRI-Welcome Trust and KEMRI-Walter Reed Program (currently known as USAMRD-A) [22]. It was also the rotavirus-testing platform of choice for various surveillance studies including the African Rotavirus Surveillance Network among others [12, 23]. EIAs have proven to be reliable in Kenya making them good tool to compare newer multiplex platforms that are emerging in the country.

This study compared a multiplex RT-PCR assay, Seeplex® Diarrhea-V ACE Detection (Seegene, Seoul, Republic of Korea), capable of detecting five enteric viruses (rotavirus, adenovirus, astrovirus, norovirus GI and GII) [24] to the established singleplex EIA, Premier™ Rotaclone® (Meridian Bioscience, Cincinnati, Ohio), that has been previously used for several rotavirus surveillance studies in well-funded research institutions in Kenya [25]. Even though Premier™ Rotaclone® has been used as a reference assay for an evaluation study done in Kenya [26], there is paucity of information on its evaluation and performance in the country. A study done in Niger in 2017 reported the Premier™ Rotaclone® to have a sensitivity of 80.7% (95% confidence interval 72.4–87.3%) and specificity of 100% (95% confidence interval 97.2–100%) [27]. Similarly there is paucity of information regarding evaluation and performance of Seeplex® Diarrhea-V ACE Detection kit in Kenya, an evaluation and verification study done in Canada reposted specificity and sensitivity of 100% for rotavirus [24]. This study sought to determine the suitability of the newer multiplex Seeplex® Diarrhea-V ACE Detection kit over the established Premier™ Rotaclone® kit by evaluating turnaround time (TAT), sensitivity, specificity and average cost. We hypothesized that there is no difference in the performance and cost of the 2 assays.

Real-time PCR (qRT-PCR) platforms detect products on-site by measuring fluorescence on each well making it more sensitive than the ethidium bromide-based detection method used by many conventional RT-PCRs kits including the Seeplex® Diarrhea-V ACE Detection kit. Unlike conventional RT-PCR that detects products at the plateau stage of the reaction, qRT-PCR detects products at the exponential stage thus it is less vulnerable to product degradation at later stages [28]. qRT-PCR also excludes ambiguity of positive/negative interpretation, which is a common issue with conventional RT-PCR products as faint bands may be difficult to interpret and can easily introduce bias [28, 29]. Because of its higher sensitivity and superior performance, qRT-PCR was used as the reference assay in this study.

This study seeks to address the paucity of performance information regarding enteric viruses’ detection assays using samples from Kenya and other developing countries.. Findings from this study will be useful in elucidating the best testing platforms to employ for the detection of rotavirus in routine clinical and research settings in Kenya and other developing countries. This will help policy makers and other stakeholders in the health sector make more informed decisions regarding rotavirus testing platforms to employ in different settings and situations.

## Materials and methods

### Study participants

Stool samples tested in this study were obtained from participants enrolled in the ongoing EPS study in Kenya. Based on the EPS study protocol, we enrolled acute, uncomplicated diarrhea cases and asymptomatic age-matched controls of all ages in several outpatient departments of various Ministry of Health (MoH) facilities in Kenya. A total of 125 stool samples collected from subjects enrolled into the EPS study from April 2013 to January 2018 used in this study. The samples were stored at − 80 °C in monitored freezers prior to testing; no preservatives were added to the samples.

### Inclusion criteria

Patients presenting with acute diarrhea defined as having 3 or more loose/watery stools within a 24-h period, lasting less than 14 days in duration and without antibiotic use were enrolled in the study as cases at the outpatient clinic of each sentinel site. Patients visiting outpatient departments of the same hospitals, whom had not had diarrhea within the previous 2 weeks, were enrolled as age-matched controls for the cases.

### Exclusion criteria

Individuals with chronic diarrhea (lasting more than 2 weeks), those who had taken antibiotics, those admitted into inpatient departments and those unwilling to provide informed consent were excluded from the study.

### Study location

This study was conducted at the United States Army Medical Research Directorate-Africa (USAMRD-A) Microbiology Hub Kericho (MHK) Kenya. The MHK conducts research in collaboration with the Kenya Medical Research Institute (KEMRI) and other institutions on the etiology of diarrheal disease in Kenya and receives samples from various government and military hospitals/clinics within Kenya.

### Scientific and ethical review

The Surveillance of Enteric Pathogens Causing diarrheal illness in Kenya (WRAIR # 1549/ KEMRI SCC# 1549) (EPS) study was approved by the KEMRI and the Walter Reed Army Institute of Research (WRAIR) institutional review boards (IRBs). This comparison of diagnostic methods study was conducted as a sub-study of the EPS study and was approved by the KEMRI and WRAIR IRBs and designated as WRAIR #2443 and KEMRI/SERU/CCR/0052/3384.

### Laboratory analysis of stool samples

#### Rotavirus testing by the EIA

The Premier™ Rotaclone® kit (Meridian Bioscience, Cincinnati, Ohio, U.S.A) was used according to the manufacturer’s instructions. Briefly, a 1:10 dilution of stool sample was prepared by adding 1 ml/1 g of stool and 10 ml of sample diluents to a well. The diluted sample was mixed with the enzyme conjugate, incubated at room temperature for 60 min, carefully poured off and wells washed five times with sterile deionized water. Enzyme substrates were added to each well and incubated at room temperature for 10 min. A total of 100 μl of stop solution was added followed by visual determination of reactivity relative to positive and negative controls provided by the kit manufacturer.

#### Rotavirus detection by RT-PCR

The ZR Soil/Fecal RNA Microprep™ kit (Zymo Research, California, U.S.A) was used for RNA extraction according to the manufacturer’s instructions. Quality and quantity of the extracted RNA was measured at 260 and 280 nm using a NanoDrop™ 2000 Spectrophotometer, a concentration > 1.8 was considered good enough for subsequent steps. Reverse transcription was done using the RevertAid First Strand cDNA Synthesis kit (Thermo Scientific, Vilnius, Lithuania) according to the manufacturer’s instructions. Synthesized cDNA was amplified using the Seeplex® Diarrhea-V ACE Detection kit (Seegene, Seoul, Republic of Korea) according to the manufacturer’s instructions. Amplicons were visualized using Ultraviolet irradiation after electrophoresis on a 3% agarose gel. Positive, negative and internal controls provided by the kit manufacturer were included in every run.

#### Resolution of discordant samples by real time RT-PCR (qRT-PCR)

qRT-PCR platform has been documented to have better performance than EIAs and conventional RT-PCR due to better detection of PCR products and reduced ambiguity in negative and positive samples [28, 29]. For these reason a qRT-PCR platform (Rotavirus/Norovirus/Astrovirus Real-Time kit by Sacace™ Biotechnologies, Como, Italy) was used as the reference assay in this study. The kit was used according to the manufacturer’s instructions. PCR cycling parameters were as follows: 2 holding stages at 50°C and 90°C for 30 min and 15 min respectively followed by 45 cycles at 95°C for 10 s, 60°C for 25 s and 72°C for 10 s. A Ct value lower than 33 was considered positive as instructed by the kit manufacturer.

### Statistical analysis

#### Sensitivity and specificity

Sensitivity and specificity were calculated as described by R. Parikh [30]. Even though Premier™ Rotaclone® is more established and often used as a reference assay, it has displayed varying sensitivities and specificities in different settings. Its sensitivity and specificity are 100 and 92% respectively according to the manufacturer while studies done in different settings have reported sensitivities of 76.8 to 80.7% in U.S.A and Niger respectively. Both studies reported 100% specificity [27, 31]. For this reason we sought to determine its sensitivity alongside that of the Seeplex® Diarrhea-V ACE Detection kit in the Kenya setting. A qRT-PCR assay was used as a reference assay.

### Turnaround time (TAT)

In this study, TAT was defined as the time taken from the beginning of sample processing to obtaining and validating the results [32]. TATs per run for both methods were recorded and used to find the mean TAT for each assay and the difference in means using the Student’s t-test.

### Determination of average cost

Information on input for materials and unit costs associated with rotavirus testing by each assay such as cost of reagents and disposable supplies including test kits were considered [33]. The average cost per test by each assay was determined by dividing the total cost of each assay by the total number of samples tested. Due to high level of variability in prices of laboratory equipment depending on manufacturers and capabilities, those that are required by the assays evaluated in this study were only listed for readers’ consideration.

## Results

### Sensitivity, specificity, predictive values and diagnostic accuracy

A total of 125 stool samples were tested in this study. Premier™ Rotaclone® kit detected rotavirus in 11.2% (14/125) while Seeplex® Diarrhea-V ACE Detection kit detected rotavirus in 16.0% (20/125). Seeplex® Diarrhea-V ACE Detection kit detected rotavirus in 25.4%, (16/63) of cases and 6.5% (4/62) of the controls. On the other hand, Premier™ Rotaclone® kit detected rotavirus in 22.2% (14/63) of cases and in none of the controls as shown in Tables 1 and 2. Discordant samples noted were from 4 controls and 2 cases, resolution by qRT-PCR revealed that the 2 cases and 2 of the controls were true positives while the other 2 controls turned out to be false positives. No cross-reactivity was observed in any of the assays. Sensitivity and specificity were calculated after resolution of discordant samples by qRT-PCR.

**Table 1:** Distribution of Rotavirus among study participants

	Subjects ≤5 yrs. of age (*n* = 64)		Subjects > 5 yrs. of age (*n* = 61)		Total
	Males (*n* = 33)	Females (*n* = 31)	Males (*n* = 27)	Females (*n* = 34)	
Cases	7	6	0	0	13
Controls	2	0	2	1	5
Total	9	6	2	1	18

**Table 2:** Rotavirus detection frequency and descriptive metrics for the two assays

	Detection frequency (%)	
	Seeplex® Diarrhea-V ACE Detection(*n* = 125)	Premier™ Rotaclone® (n = 125)
Cases *n* = 63	16/63 (25.4%)	14/63 (22.22%)
Controls *n* = 62	4/62 (6.4%)	0/62 (0)
Total = 125	20/125 (16.0%)	14/125 (11.2%)

The sensitivity and specificity of the Seeplex® Diarrhea-V ACE detection were 100% (95% confidence interval 81.5 to 100.00%) and 98.1% (95% confidence interval 93.41 to 99.77%) respectively, and those of the Premier™ Rotaclone® were 77.8% (95% confidence interval 52.36 to 93.59%), and 100% (95% confidence interval 96.61 to 100.00%) respectively as shown in Table 3

**Table 3:** Comparison of the sensitivity and specificity of the Seeplex® Diarrhea-V ACE Detection and Premier™ Rotaclone® assays

	Seeplex® Diarrhea-V ACE Detection (n = 125)	Premier™ Rotaclone® (n = 125)
True positives	18	14
False positives	2	0
True negatives	105	107
False negatives	0	4
Sensitivity	100% (95% CI: 81.5 to100%)	77.8% (95% CI: 52.36 to 93.59%)
Specificity	98.1% (95% CI: 93.41 to99.77%)	100% (95% CI: 96.61 to 100.00%)

### Turnaround time (TAT)

The mean TAT per run of 10 samples was 93 min and 359 min for the Premier™ Rotaclone® and the Seeplex® Diarrhea-V ACE Detection respectively (Table 3). The difference in mean TATs was 266 min (95% confidence interval from 262 to 270 min). The Seeplex® Diarrhea-V ACE Detection kit takes more than threefold the amount of time it takes to run the Premier™ Rotaclone®.

### Cost

The average cost of kits/reagents used for testing one sample was 13.33USD and 32.94USD for the Premier™ Rotaclone® and the Seeplex® Diarrhea-V ACE Detection assay respectively (Table 4). The Premier™ Rotaclone® only required one kit, a pipette, pipette tips, and sterile tubes while the RT-PCR assay required 2 additional kits (an RNA extraction and a reverse transcription kit), as well as Thermo Scientific™ NanoDrop™ 2000 Specrtophotometer~ 9700USD, a thermocycler (Applied Biosystems Veriti 96 well thermocycler ~7000USD), high-speed centrifuge (Eppendorf Microcentrifuge ~3000USD), an electrophoresis power supplier (Thermo Scientific Owl EC-200 XL Hi Current Power supply~ 2000USD), an electrophoresis chamber (Thermo Scientific Owl A1 Large Gel System~650USD), and a gel imaging system (Alpha Innotech AlphaImager HP~ 2500 USD). Unlike consumable reagents and test kits that are bought frequently and have predictable prices, laboratory equipment are not bought frequently and vary greatly in prices depending on their capabilities and vendors. For these reasons, cost of equipment used in this study are only listed for readers’ consideration but not included alongside consumable reagents and kits in calculation of the average cost per sample represented in Table 4.

**Table 4:** Summary of TAT and average reagent and kits cost per assay

	Seeplex® Diarrhea-V ACE Detection	Premier™ Rotaclone®
Mean TAT (per run of 10 samples)	359	93
Average cost (per sample)	$32.94	$13.33

### Additional findings

Due to the multiplex nature of the Seeplex® Diarrhea-V ACE Detection kit, we detected a variety of enteric viruses including a 1 subject who had a rotavirus/norovirus co-infection (Table 5).

**Table 5:** Enteric viruses detected by the Seeplex® Diarrhea-V ACE Detection Assay

	Subjects ≤5 yrs. of age (*n* = 64)		Subjects > 5 yrs. of age (*n* = 61)		Total
	Males	Females	Males	Females	
Rotavirus	9^a^	6	2	3	20
Adenovirus	3	2	0	1	6
Astrovirus	1	0	0	0	1
NoVGI	0	0	0	0	0
NoVGI1	3^a^	5	2	2	12
Total	16	23	4	6	39

^a^1 case of rotavirus-norovirus GII co infection

## Discussion

In this study, the Seeplex® Diarrhea-V ACE Detection kit showed a higher detection frequency and was more sensitive than the Premier™ Rotaclone®. The two kits had detection frequencies of 25.4 and 22.2% respectively among cases, all of these were children below 5 years of age. These resulted closely reflects prevalence of 14.5 to 31% that were reported in recent epidemiologic studies done in Kenya among children below 5 years of age hospitalized with diarrhea [10, 12]. The higher detection frequency and sensitivity of the RT-PCR assay could be attributed to its lower detection limit documented by the manufacturer (100 copies/3 μl DNA) that enables it to detect trace amounts of viral nucleic acids excreted in stool samples. The observation that rotavirus was detected in asymptomatic controls by RT-PCR could be attributed to the fact that the virus’ nucleic acid is likely to remain detectable for a longer period after the subjects has recovered. In contrast, the EIA targets antigens that are rarely detected more than 1 week after onset of illness [34]. Asymptomatic controls have also tested positive by RT-PCR in other studies including one recently done in Niger [27]. The higher sensitivity (100%) of the Seeplex® Diarrhea-V ACE Detection kit was observed in an evaluation and verification study done in Canada [24]. The higher specificity (100%) exhibited by Premier™ Rotaclone® in this study is also in agreement with previous studies comparing EIA and RT-PCR detection assays U.S.A [31].

The mean TAT per run of 10 samples for Seeplex® Diarrhea-V ACE Detection kit was more than three-fold longer than the TAT for Premier™ Rotaclone®. This was due to several steps involved in the RT-PCR assay that include RNA extraction, reverse transcription, and amplification. This difference in mean TATs is an important consideration in clinical settings where shorter TATs are of great importance for patient treatment and care [18]. It is important to note that the Seeplex® Diarrhea-V ACE Detection kit is able to detect five different diarrhea causing viruses (rotavirus, adenovirus, astrovirus, norovirus GI and GII) within the time noted whereas the Premier™ Rotaclone® can only detected rotavirus. Seeplex® Diarrhea-V ACE Detection kit was able to detect norovirus GII in 12 samples (8 from children below 5 years and 4 from individual above 5 years), adenovirus in 6 samples (5 from children under 5 years and 1 from a 6 years old child), 1 astrovirus from a 5 years old child and one rotavirus/norovirus GII co infection from a 1 year and 4 months old child. This gives a hint of the heavy disease burden imposed especially on children below 5 years old by enteric viruses circulating in Kenya. As observed by Goldenberg et al. 2015, the ability to detect multiple pathogens is an important consideration to make when identifying platforms to use for future clinical as well as outbreak investigations and surveillance work [35].

The average cost of testing one sample by Seeplex® Diarrhea-V ACE Detection was more than 2-fold higher than that of testing by the Premier™ Rotaclone®. The higher cost can be attributed to the fact that the RT-PCR assay required an RNA extraction, reverse transcription as well as amplification kits whereas the Premier™ Rotaclone® procedure only required one kit. Seeplex® Diarrhea-V ACE Detection also utilized more laboratory equipment: a spectrophotometer, thermocycler, a high-speed centrifuge, an electrophoresis power supplier, an electrophoresis chamber, and a gel imaging system making its operational cost much higher than the Premier™ Rotaclone®. The Premier™ Rotaclone® required fewer supplies due to fewer steps involved in its procedure. The RT-PCR platform had longer steps with both RNA extraction and agarose gel electrophoresis that required more supplies. This made the Seeplex® Diarrhea-V ACE Detection assay more labor intensive and time consuming than the EIA platform [36].

Accurate diagnosis and surveillance of rotavirus and other enteric viruses causing diarrhea in Kenya is critical for the rapid identification of infected patients who are potential sources of infection to others. Having this capability at the point-of-care or as close as possible would enhance patient management, reduce the spread of enteric pathogens, and improve the management of disease burden, especially among children [37]. Evaluation studies like this will ensure quality and improvement of enteric viruses’ testing assays, implementation of better testing platform, improved knowledge on disease dynamics and better patient care. These outcomes will have a beneficial effect especially on the health of children in Kenya and other developing countries that bear the biggest disease burden associated with enteric viruses. This will lead to better growth, development and general well being of such children.

## Conclusion

In this study, the Seeplex® Diarrhea-V ACE Detection kit (Seegene, Seoul, Republic of Korea), a RT-PCR, showed a higher detection frequency and sensitivity compared to the established EIA, Premier™ Rotaclone® kit(Meridian Bioscience, INC, Cincinnati Ohio, U.S.A). However, the Premier™ Rotaclone® kit had a higher specificity, lower average cost, and shorter TAT.

With a higher detection frequency, sensitivity, and ability to detect 5 viruses, the Seeplex® Diarrhea-V ACE Detection kit is an ideal tool for outbreak investigations and surveillance of enteric viruses causing diarrhea in Kenya. Its higher cost, complexity, and longer TAT make it unsuitable for routine clinical testing especially in austere setups within Kenya and other developing nations. However, if validated and approved by the relevant authorities in Kenya, it can be a useful tool in well-funded clinical laboratories within the country. This platform can be easily implemented in well-funded research institutions in Kenya and can play an important role in elucidating the incidences, prevalence, distribution, and disease burden associated with enteric viruses circulating in the country. The longer TAT and higher cost of the Seeplex® Diarrhea-V ACE assay may be justifiable by the number of viruses it can detect in each run.

The Premier™ Rotaclone® is suitable for routine clinical testing of rotavirus especially in austere settings in Kenya. It has a shorter turnaround time, is 100% specific, cheaper and can be easily implemented even in remote clinics that have limited resources and financial constraints that inhibit their ability to implement complex platforms like RT-PCR.

Further evaluation and validation of newer multiplex assays that can detect several enteric pathogens including parasites and bacteria circulating in Kenya should be encouraged, as this will elucidate their applicability and make scientists and policy makers aware of the most suitable platforms to implement in routine clinical and research work in Kenya. The additional data that multiplex assays are able to provide can enhance surveillance of etiological agents causing diarrheal illness and enhance public health measures and policy decisions on diarrheal diseases. Other than rotavirus, there is very limited information on the prevalence and trends of other enteric viruses circulating in Kenya. As rotavirus studies continue, more effort should be put on studying the other enteric viruses as well. This should be done by the most suitable testing platforms that can be implemented efficiently to generate the most accurate data possible.

### Limitations of the study

Limitations of this study include the fact that we did not look at severity of illness and focused only on 5 enteric viruses thus we cannot provide information on other pathogens including bacteria and parasites that are the other likely causes diarrhea in the cases that were negative for the enteric viruses tested in this study.

## Data Availability

All data and material used to generate information published in this paper shall be made available to publisher or any relevant party that may need the information.

## Abbreviations

CAP: College of American Pathologists
dNTPs: Deoxyribonucleotide triphosphate
EIA: Enzyme immunoassay
EM: Electron microscopy
EM: Electron microscopy
IRB: Institutional Review Board
KEMRI: Kenya Medical Research Institute
MHK: Microbiology Hub Kericho
MoH: Ministry of Health
mRT-PCR: Multiplex reverse transcription polymerase chain reaction
PCR: Polymerase chain reaction
qRT-PCR: Real-time reverse transcription Polymerase chain reaction
TAT: Turn around time
UKNEQAS: United Kingdom National External Quality Assessment Service
USAMRD-A: United States Army Medical Research Directorate-Africa
WHO: World Health Organization
WRP: Walter Reed Project
